# Supplementary material for: Efficacy of mecobalamin (vitamin B12) in the treatment of long-term pain in women diagnosed with fibromyalgia: protocol for a randomised, placebo-controlled trial
Source: BMJ Open. 2023 Mar 30;13(3):e066987. doi: 10.1136/bmjopen-2022-066987 (PMC10069488; doi:10.1136/bmjopen-2022-066987)
Supplement: Supplementary data [file bmjopen-2022-066987supp001.pdf]

Participant informed consent form

I have been informed orally about the study and I have read the written information. I have received answers to my questions and I agree to participate in the study. My participation is voluntary, and I am aware that I can withdraw without any explanation and without affecting my future care.

I am informed about my rights to get register extract once a year over collected data. I have been informed about my rights to get false information's arranged or removed from the register. I have been informed about samples taken in the study are handled according to the Biobank Act. I am aware that my sample material will be destroyed after analyses.

Consent regarding data management

I have received information that personal data collected in the study will be handled confidentially, which means my identity will not be revealed to unauthorized persons.

I allow an independent person and/or authority person, appointed by the study management, to review study information provided customary confidentiality are maintained.

We ask you to confirm your identity at all occasions with legitimation.

……………………………. ………………………………….

Participants' signature Date (written by participant)

……………………………..

Name clarification

………………………………. …………………………………

Physician signature Date

…………………………………

Name clarification

☐ Signed copy of the information and consent has been distributed to the participant.
