# Supplementary material for: Efficacy of mecobalamin (vitamin B12) in the treatment of long-term pain in women diagnosed with fibromyalgia: protocol for a randomised, placebo-controlled trial
Source: BMJ Open. 2023 Mar 30;13(3):e066987. doi: 10.1136/bmjopen-2022-066987 (PMC10069488; doi:10.1136/bmjopen-2022-066987)
Supplement: Author's manuscript [file bmjopen-2022-066987.draft_revisions.pdf]

BMJ Open is committed to open peer review. As part of this commitment we make the peer review history of every article we publish publicly available.

When an article is published we post the peer reviewers' comments and the authors' responses online. We also post the versions of the paper that were used during peer review. These are the versions that the peer review comments apply to.

The versions of the paper that follow are the versions that were submitted during the peer review process. They are not the versions of record or the final published versions. They should not be cited or distributed as the published version of this manuscript.

BMJ Open is an open access journal and the full, final, typeset and author-corrected version of record of the manuscript is available on our site with no access controls, subscription charges or pay-per-view fees (http://bmjopen.bmj.com).

If you have any questions on BMJ Open's open peer review process please email info.bmjopen@bmj.com

BMJ Open

Evaluation of the efficacy of Mecobalamine (Vit B12) in the treatment of long-term pain in women diagnosed with fibromyalgia: Randomized controlled trial protocol

Journal:	*BMJ Open*
Manuscript ID	bmjopen-2022-066987
Article Type:	Protocol
Date Submitted by the Author:	04-Aug-2022

Complete List of Authors:	Sall Hansson, Karin; Linnaeus University Faculty of Health and Life Sciences, Dept. of Health and Caring Sciences, Linnaeus University<br>Lindqvist, Gunilla; Linnaeus University Faculty of Health and Life Sciences, Dept. of Health and Caring Sciences, Linnaeus University<br>Stening, Kent; Linnaeus University Faculty of Health and Life Sciences, Dept. of Health and Caring Sciences, Linnaeus University<br>Fohlman, Jan; Region Kronoberg<br>Wojanovski, Anna; Region Kronoberg<br>Ponten, Moa; Karolinska Institute<br>Jensen, Karin; Karolinska Institute<br>Gerdle, Björn; Linkopings universitet, Pain and Rehabilitation Centre, and Department of Medical and Health Sciences<br>Elmqvist, Carina; Linnaeus University Faculty of Health and Life Sciences, Dept. of Health and Caring Sciences, Linnaeus University; Linnaeus University Faculty of Health and Life Sciences, Centre of Interprofessional Cooperation within Emergency care (CICE)

Keywords:	COMPLEMENTARY MEDICINE, PAIN MANAGEMENT, QUALITATIVE RESEARCH, STATISTICS & RESEARCH METHODS, Clinical trials < THERAPEUTICS

SCHOLARONE™
Manuscripts

Evaluation of the efficacy of Mecobalamine (Vit B12) in the treatment of long-term pain in women diagnosed with fibromyalgia: Randomized controlled trial protocol

Karin Säll Hansson [1 2]
RN, MScN, lecturer, Department of Health and Caring Sciences, Linnaeus University Växjö, Sweden

Gunilla Lindqvist [1 2]
RN, PhD, Senior lecturer, Department of Health and Caring Sciences, Linnaeus University, Växjö, Sweden.

Kent Stening [1 2]
RN, PhD, Senior lecturer, Department of Health and Caring Sciences, Linnaeus University, Kalmar, Sweden

Jan Fohlman [1 2]
MD, PhD, Associate Professor, Region Kronoberg, Sweden

Anna Wojanowski [1 2]
MD, Region Kronoberg, Sweden

Moa Pontén, [3]
MSc, Doctoral student, Department of Clinical Neuroscience, Karolinska Institutet, Sweden

Karin Jensen, [3]
Associate Professor, Department of Clinical Neuroscience, Karolinska Institutet, Sweden

Björn Gerdle, [4]
MD, PhD, Professor emeritus, Department of Health, Medicine and Caring Sciences, Linköping University, Linköping, Sweden

Carina Elmqvist, [1 2]
RN, PhD, Associate Professor, Department of Health and Caring Sciences, Linnaeus University, Växjö, Head of Research, Region Kronoberg, Sweden

[1]Pain Research group, Linnaeus university
[2]Centre of Interprofessional Collaboration within Emergency care (CICE), Linnaeus university
[3]Neuroimagine lab, Karolinska Institute
[4]Pain and Rehabilitation Center, Linkoping, Sweden

Corresponding author: Carina Elmqvist carina.elmqvist@lnu.se
RN, PhD, Associate Professor, Department of Health and Caring Sciences, Linnaeus University, Växjö, Head of Research, Region Kronoberg, Sweden

For peer review only - http://bmjopen.bmj.com/site/about/guidelines.xhtml

ABSTRACT

Introduction

Fibromyalgia causes long term pain. It affects at least 2% of the population, the majority being women. In addition, extended symptoms corresponding to vitamin B12 deficiency occur. Primary studies indicate interaction effects between the receptor of NMDA which is involved in both long-term pain and vitamin B12 deficiency. The aim of the proposed study is to evaluate whether vitamin B12 decreases pain sensitivity and the experience of pain i.e. hyperalgesia and allodynia in women suffering of fibromyalgia.

Methods and analysis

The study is a randomised placebo-controlled (RCT), single-blind, clinical trial with two parallel groups who are administered Mecobalamin (vitamin B12) v/s placebo over 12 weeks. Outcomes consist of questionnaires and QST measured at baseline and after 12 weeks of treatment. A final re-evaluation will then follow 12 weeks after treatment ends. In order to broaden the understanding of the lived experience, an interview is conducted using a phenomenological approach on a life-world theoretical basis, the Reflective Lifeworld Research approach (RLR).

Ethics and dissemination

The protocol for the study is approved by the local ethical committee at Linkoping (EPM); 2018/294-31 (Appendix 2019-00347; 2020-04482), and the Swedish Medical Products Agency; EudraCT-no 2015-005086-23 (Appendix 5.1-2020-71076). The study started 6 of February 2019, recruiting is delayed caused by COVID-19. Trial results expects during 2024 and will primarily be communicated through peer-reviewed journals and conferences.

Title registration: EUDRACT 2015-005086-23

ClinicalTrials.gov: ID NCT05008042

Keywords: Fibromyalgia, Cobalamin, Chronic pain, Mekobalamin, Randomised controlled trial, Vitamin B12

For peer review only - http://bmjopen.bmj.com/site/about/guidelines.xhtml

STRENGHT AND LIMITATION OF THIS STUDY

- The trial is a collaboration between different universities and regions.
- To the best of our knowledge, this is the first trial that specifically examined the effect of Mecobalamin (Vitamin B12) on pain in the current patient group, women diagnosed with fibromyalgia.
- The recruitment period will likely be delayed by COVID-19.

INTRODUCTION

Fibromyalgia causes long-term pain and affects at least 2% of the population, 80% of the sufferers being women (1). Treatment options are scarce and patients with fibromyalgia have experienced being discredited and invalidated by the health-care system (2). Fibromyalgia is characterized by long-term widespread musculoskeletal pain and generalized hyperalgesia. This is often accompanied by fatigue, concentration problems and sleep problems (3). Fibromyalgia pain is currently classified as nociplastic, which means that pain arises from altered regulation of pain signals, in absence of tissue damage (4).

No definite pathophysiology has been established for fibromyalgia. Imaging techniques have challenged previous ideas about the peripheral origin of FM and have provided evidence for altered central nervous system (CNS) nociceptive/pain processing and morphology in fibromyalgia. Furthermore, recent studies report both central alterations and peripheral alterations (e.g., systemic low-grade inflammation and nociceptor/muscle alterations) (5-9)

The treatment for Fibromyalgia includes both non-pharmacological and pharmacological interventions, depending on the key symptoms and the extent of disability. The recommended pharmacological treatments for Fibromyalgia are antidepressants (e.g. SNRI) and antiepileptics (e.g. gabapentinoids), which often bring side effects (4). In addition, opioid analgetics and NSAID/COX are often prescribed (10). However, only a

For peer review only - http://bmjopen.bmj.com/site/about/guidelines.xhtml

For peer review only

minority of individuals report a clinically relevant improvement from any of the treatments (11).

Vitamin B12 is sometimes used for symptoms other than those of Vitamin B12 deficiency for example, different pain conditions such as backpain and neuropathic pain (12-16). Vitamin B12 nasal drops have also been tested and shown positive results on patients with ME/CF/fibromyalgia (myalgic encephalomyelitis/chronic fatigue syndrome) (17). In addition, there are Vitamin B12 studies that have shown good results in the therapy of aphthous ulcers (18) and acute lumbago (19) as well as in studies concerning diabetic polyneuropathy (20, 21). Moreover, there are studies that examined methylcobalamin treatment on patients with lower back pain showing pain reduction and functionality gain (13, 22).

To summarize, it is not entirely clear how Vitamin B12 affects the human pain system. However, several studies indicate that it may be a possible treatment in fibromyalgia.

Aim and objectives

The aim of this study is to evaluate the effect of Mecobalamin (Vitamin B12), and describe lived experiences of pain, health, suffering and well-being in women with diagnosed fibromyalgia.

The primary objective is to evaluate whether Mecobalamin (Vitamin B12) given as an intramuscular injection once/week for 12 weeks compared to placebo reduces pain sensitivity i.e. tolerance time (Cold Pressor Test).
The secondary objective is to evaluate whether intramuscular Mecobalamin (Vitamin B12) compared to placebo reduces pain intensity and pressure pain threshold (Numeric rating scale NRS, Pressure Algometry), increases activity level (questionnaire), quality of life (questionnaire) and perceived effect of given drug (questionnaire). Furthermore, the ratings of expected effects of a given drug (NRS), the desire for pain relief (NRS) and estimated pain variability (NRS) are evaluated. Qualitative interviews that describe how

For peer review only - http://bmjopen.bmj.com/site/about/guidelines.xhtml

women with fibromyalgia experience pain, health, suffering and well-being (interviews) will be conducted.

METHODS AND ANALYSIS

Study design

The study is a randomised placebo-controlled (RCT) single-blind clinical trial using parallel groups. Participants are individually randomised to intervention or control group (placebo) in 1:1 allocation. In order to broaden and deepen the understanding of the lived experience, an interview will be conducted applying a phenomenological approach on a life-world theoretical basis, Reflective Lifeworld Research approach (RLR) (23).

Patient and public involvement

Patients and/or the public were not involved in the design, or conduct, or reporting, or dissemination plans of this research.

Study sample

Women aged 20-70 years with an earlier recorded diagnosed fibromyalgia. Detailed eligibility criteria are presented in Table 1.

Table 1. Inclusion and exclusion criteria

**Inclusion criteria:**	**Exclusion criteria:**
- Diagnosis of fibromyalgia<br>- Women aged 20-70 years<br>- Swedish-speaking<br>- Safe method of contraception<br>- Cobalamin value >250 pmol/L < 800 pmol/L<br>- Kidney function value (relatively) GFR >60 mL/min/1.73 m^2<br>- Liver function value P-ALP 0.6-2.85 µkat/L<br>P-ALAT 0.15-1.13 µkat/L<br>- Heart function value Troponin-T < 15 ng/L<br>NT-pro-BNP<br>< 60 year <125 ng/L	- Previous treatment with B12<br>- Known hypersensitivity to the active substance Mecobalamin or an additive<br>- Neuroleptic treatment<br>- Reynaud's phenomenon<br>- Known neuropathy<br>- Vegan as veganism can lead to B12 deficiency<br>- Known heart, kidney or liver disease<br>- Breastfeeding<br>- Planned or ongoing pregnancy

For peer review only

For peer review only - http://bmjopen.bmj.com/site/about/guidelines.xhtml

NT-pro-BNP<br>> 60 year < 300 ng/L<br>- Given consent to participate	

The participants are recruited via advertising in the local newspaper Facebook, YouTube, Fibromyalgia Association, posters at Linnaeus University, hospitals and healthcare centres. The prospective participant contacts the Trial Manager via telephone or email and receives oral information. Written information and consent form are sent to the participant who contacts the Trial Manager for an appointment.

Intervention and placebo to be measured

The active substance of Vitamin B12 given in the study is Mecobalamin 5mg/ml 2 ml i.e. 10 mg intramuscularly (24). Placebo substance is Sodium Chloride (NaCL) 9 mg/ml 2 ml, isotonic solution for parenteral use (Baxter) intramuscularly (25). All participants are informed that their urine may turn red, which may occur during Mecobalamin injections.

Measurements

At the first measurement opportunity, the trial manager and co-examiner 1 carefully goes through inclusion and exclusion criteria. Oral information is given again, and consent is signed by the participant and by the co-examiner 1. First, the participant answers two questionnaires Short-form McGill Pain Questionnaire (MPQ) (26) and RAND 36 (27), then stimulation tests (Cold Pressor Test, pressure algometry) (28 - 32) are performed followed of pain rating (NRS) (33) immediately, after one minute and after three minutes. Finally, blood samples for cobalamin (Vitamin B12), kidney, liver and heart function are taken. The participant provides a wrapped information card about the study's design, purpose, treatment options and contacts with telephone numbers. The participant is asked about interest in an interview in connection with the final measurement opportunity. Co-examiner 2 assesses the blood samples and approves final inclusion. If tests deviate from accepted reference values, the participant is excluded and encouraged to seek medical attention.

For peer review only

For peer review only - http://bmjopen.bmj.com/site/about/guidelines.xhtml

After approved inclusion, an independent chief examiner performs the randomisation. The participant then receives intramuscular injections of the active substance or placebo, once a week for 12 weeks, given by a registered nurse. Before each injection the participant answers questions in which they estimate their expected effects of a given drug, desire for pain relief and average pain intensity during the past week (NRS). The participant has the opportunity to postpone the visit ± 3 days, for example in case of illness or travelling and may miss a maximum of two injections in order to follow per protocol analysis. Even if the participant is not compliant with the study protocol, the participant may retain the treatment regime for the duration of the study and be analysed in Intention to Treat (ITT).

The second measurement takes place after 12 weeks of intervention and three questionnaires (MPQ, RAND 36) and (Patient Global Impression of Change PGIC) (34) are filled in. The stimulation tests Cold Pressor Test and pressure algometry are performed as well as blood sampling of cobalamin/p (Vitamin B12). In cases of low cobalamin/p, the participant is encouraged to seek medical attention but is still included in the study.

At the third measurement, the follow-up, which takes place 12 weeks after the end of the intervention, the participant fills in questionnaires (MPQ, RAND-36 and PGIC), undergoes stimulation tests (Cold Pressor Test and pressure algometry), blood sampling of cobalamin/p (Vitamin B12) together with an interview for those who so desire. If cobalamin/p on this occasion shows that the participant has a low cobalamin value, the participant is encouraged to seek medical attention. For the individual participant, the study ends after six months. An overview of the study flow is presented in Fig.1.

Please insert Figure 1 here

For peer review only - http://bmjopen.bmj.com/site/about/guidelines.xhtml

Outcomes

The primary outcome is

- Tolerance time, maximised to three minutes, which is tested using the Cold Pressor Test

The secondary outcome are;

- Pain experience and possible pain reduction measured by a pressure algometry test performed on the shoulder, hip, knee and elbow.
- The pain intensity measured by NRS.
- Ratings of expectation, desire for pain relief, and pain variability using NRS.
- Activity level and quality of life assessed using questionnaires MPQ and RAND-36.
- Experience of the effect of the drug assessed using questionnaire PGIC.
- Control of Vitamin B12 by measuring cobalamin in plasma.
- Qualitative in-depth interviews conducted to capture women's lived experiences of pain, health, suffering and well-being.

Cold Pressor Test

The Cold Pressor Test (28-30) is measured in number of seconds the participant leaves her hand in the low-temperature water of five degrees. Evaluation variables from Cold Pressor Test become pain threshold (when it starts to hurt) and tolerance time, i.e. how long the participant endures the pain. Participants end the test by raising their hands when they can no longer stand it or when the set end time of three minutes has elapsed.

Pressure algometry test

The pressure algometry test (31, 32) generates a mechanical pressure against specific points on the body; trapezius (shoulder), epicondyle (elbow), gluteal (outside the gluteal muscle) and medial knee (inside of the knee) which are all accepted in the diagnosis of fibromyalgia. Pressure algometry tests are measured in kilopascal (kPa). The Trial Manager interrupts the mechanical pressure when the participant expresses pain.

For peer review only - http://bmjopen.bmj.com/site/about/guidelines.xhtml

Numeric rating scale (NRS)

Participants rate their pain intensity on NRS (33) in connection with the Cold Pressor Test when the participant takes her hand from the water, after one minute and finally after three minutes. Participants estimate their pain intensity on a scale (NRS) from 0-10, where 0 corresponds to no pain at all and 10 worst possible pain. Pain rating is measured in the same way after the pressure algometry test.

The influence of expectation, desire for pain relief, and pain variability will be measured before each injection using the same numeric rating scale as used for pain ratings. Ratings of expected pain levels will obtained by asking patients “What level of pain do you expect when this treatment starts to have an effect?” The NRS scale is anchored at the left by the descriptors ´no pain sensation´ and at the right by ´the most pain sensation imaginable´. Ratings of desired pain relief will be obtained by asking patients “How strong is your desire for pain relief? The question will be anchored by the descriptors ‘0=No desire for pain relief’ and ‘10=the most intense desire for pain relief imaginable’. Ratings of pain variability will be obtained by asking participants their average pain intensity that week. These scales have been validated and used in previous studies (35-39).

Short-form McGill Pain Questionnaire (MPQ)

In this study MPQ Short form of 15 questions (26) is used which distinguishes between the sensory-discriminatory and the affective and emotional aspects of the pain experience. Participants are asked to estimate their pain intensity and describe their pain in predetermined words. They fill in MPQ on all three measurement occasions.

RAND-36

RAND-36 is a quality-of-life instrument (27) that aims to measure health-related quality of life, i.e. physical, mental and social well-being and not merely the absence of illness. The estimation instrument consists of 36 questions of various kinds, e.g. on general health, activity, physical health,

For peer review only - http://bmjopen.bmj.com/site/about/guidelines.xhtml

emotional problems and pain. RAND-36 is filled in by the participant on all three measurement occasions.

Patient Global Impression of Change (PGIC)

PGIC (34) evaluates participants' experience of the treatment i.e. the substance given. Participants tick the box that most closely describes the change that they are experiencing. The PGIC is completed by the participant after the injection treatment has been concluded at 12 weeks and at the follow-up 12 weeks later. PGIC is directly translated in its entirety from English by the research group.

Cobalamin/p

Cobalamin/p levels are taken on three occasions: at the first measurement, baseline, in order to rule out that none of the participants has a Vitamin B12 deficiency (according to the accepted reference value). At the second measurement, at 12 weeks on completion of treatment in order to monitor the participants' level of cobalamin/p just after the end of the injection treatment. The final measurement is12 weeks after the end of intervention.

Qualitative in-depth interview

In order to obtain a deeper understanding of women's perceived experiences, in-depth interviews with both compliant and non-compliant participants from both groups will be performed in connection with the third measurement session. The aim is to describe the women's lived experiences of pain, health, suffering and well-being. The interviews will be conducted by the Trial Manager, recorded, transcribed and later analysed using a reflective life world approach (RLR) (23).

Sample Size

In order to obtain an adequate sample size, a power analysis was performed based on results from a previously published study (28) in which women with the current diagnosis, fibromyalgia, underwent the same stimulation procedure with the Cold Pressor Test. Account is taken here of the standard deviation shown and compensation for any placebo effects. Based on these

For peer review only - http://bmjopen.bmj.com/site/about/guidelines.xhtml

results, the expected effect difference is estimated at 20 seconds with a standard deviation of 20 seconds between the two groups after 12 weeks of treatment with Mecobalamin (Vitamin B12)/placebo. Using a two-sided t-test for two independent groups, with a power of 80% and 5% significance level, it was calculated that each group would consist of 16 participants. In order to compensate for any loss, 20 participants per group are therefore included. The study continues until 40 participants have been included and completed the study.

Randomisation and allocation concealment

Participants are randomised into two groups, the placebo group or the treatment group, each consisting of 20 participants either receiving Mecobalamin (Vitamin B12) or placebo (NaCL). An independent statistician generates a randomisation list by random distribution of processing in blocks (1:1) in a computerised statistics program (STATA). Participants are randomised according to the list by the Chief Examiner opening closed randomisation envelopes in consecutive order. Based on each randomisation envelope, the Chief Examiner registers in and signs the injection retrieval protocol as to which substance the participant will receive. The participants' randomisation number is recorded in the journal. Only the Chief Examiner has access to randomisation envelopes and injection retrieval protocols which are kept locked.

When the third measurement session has been completed, the Trial Manager contacts the Chief Examiner who has the randomisation list.

The Chief Examiner breaks the code and notifies the participant by letter of which substance she has been treated with. The code is broken at this time because it cannot be considered ethical that the participant does not find out about the treatment given, especially if the participant believes that the treatment has helped. If the participant has received Mecobalamin and wishes to continue, they are recommended to contact their health centre for continued Mecobalamin treatment. The letter will contain the contact information of the Trial Manager and the Chief Examiner if the health centre doctor wishes to contact them. The Trial Manager is still unaware of what the individual participant received until the study is completed. If the code needs

For peer review only - http://bmjopen.bmj.com/site/about/guidelines.xhtml

to be broken for an individual participant before the third measurement session has been completed, there is a code breaking envelope that may be used by the Chief Examiner in cases of emergency.

Statistical methods

The primary endpoint is tolerance time at Cold Pressor Test will be calculated on the difference between the two groups after 12 weeks and will be analysed by ANOVA. Pain thresholds measured by pressure algometry test (Somedic) will be analysed using: t-test, ANOVA, similar to the primary variable. Questionnaires (MPQ, RAND-36, and PGIC) will be analysed using Mann-Whitney tests. In cases of minor loss, (less than 10%), a mixed model can be used as an alternative to t-test and ANOVA with repeated measurement. For variables with more than 10% loss, imputation of data will be used. Drop-out participants will be described in a specific analysis. Rejection analysis will be performed on the participants who interrupt the study prematurely.

The study will present results both from “intention to treat” and “per protocol” analyses. Intention to treat" is defined as the participant is placed in the treatment group she had been randomised to. "Per protocol" is defined as the participant is placed in the treatment group she de facto received and carried out without significant protocol deviations. “Per protocol” will be presented as sensitivity analysis. The participant may miss a maximum of two injections.

Self-reported ratings of desired and expected treatment outcome pre and post treatment will be implemented in linear regression models to predict any of the outcome measures. Repeated ratings of desired treatment outcome, expected treatment outcome and pain variability will be calculated as each participant's standard deviation from the repeated ratings and implemented in a linear regression model to predict possible outcome measures.

For peer review only - http://bmjopen.bmj.com/site/about/guidelines.xhtml

ETHICS AND DISSEMINATION

The protocol for the study is approved by the local ethical committee at Linkoping (EPM); 2018/294-31 (Appendix 2019-00347; 2020-04482) and the Swedish Medical Products Agency; EudraCT-no 2015-005086-23 (Appendix 5.1-2020-71076) furthermore monitored from Forum Ostergotland, Linkopings university. The study is registered at ClinicalTrials.gov: ID NCT05008042, follows Good Clinical Practice (GCP) and adheres to the principles of the Helsinki Declaration (2013) (40). All participants must give their written as well as oral consent to participate. They will be informed that they may discontinue the study whenever they wish to without giving any reason for their decision. The study started 6 of February 2019, recruiting is delayed caused by COVID-19. Trial results expects during 2024 and will be disseminated at national and international conferences, peer-reviewed journals, newsletters and magazines.

Discussion

The results of the study will investigate whether Vitamin B 12 decreases pain sensitivity and experience of pain in women suffering from fibromyalgia. The European Federation of the IASP (The international Association for the Study of Pain) (EFIC) Declaration from 2001 expressed that, *"very few people die of pain, many dies in pain and even more live in pain"*. To live in pain affects the whole human existence and people with chronic pain such as fibromyalgia often feel discredited, having to fight to assert their right to treatment and to receive the necessary care (2). Today, there is no curative treatment for fibromyalgia (41, 42). Since opioid analgesics, SNRI and gabapentinoids are the most used pharmacological treatments for Fibromyalgia, often with side effects (4, 10), there is a need for alternative treatments (11), with less side effects.

Furthermore, there is currently a lack of studies on patients' subjective experiences of living with long-term pain and fibromyalgia (43, 44), why it is important to evaluate the effect of Mecobalamin (Vitamin B12), and describe lived experiences of pain, health, suffering and well-being in women with diagnosed fibromyalgia.

For peer review only - http://bmjopen.bmj.com/site/about/guidelines.xhtml

Acknowledgements

We thank Co-examiner and Medical Doctors; Anders Willstedt and Bjarne Sorensen as well as the Registered Nurse Ann-Louise Breider for their ongoing contributions and support.

Author affiliations

[1]Pain Research group, Linnaeus university

[2]Centre of Interprofessional Collaboration within Emergency care (CICE), Linnaeus university

[3]Neuroimagine lab, Karolinska Institute

[4]Pain and Rehabilitation Center, Linkoping, Sweden

Contributors

KSH, GL, KS, JF, AWo, MP, KJ, BG and CE planned the study design and all authors participated in the production of the analysis plan. All authors participated in the acquisition of data. KSH, GL, KS and CE prepared the first version of the manuscript and all authors participated in reviewing and drafting the manuscript. All authors have read and approved the final version.

Conflict of interest

There are no conflicts of interest to report

Funding

This trial was supported by grants from Medical Research Council of Southeast Sweden no: 479961; 656241; 854911 and Capio Research Foundation, Sweden no: 2018-3195; 2019-3277. The funding sources had no involvement in study design, data collection or the writing of this manuscript.

REFERENCES

1. Heidari F, Afshari M, Moosazadeh M. Prevalence of fibromyalgia in general population and patients, a systematic review and meta-analysis. *Rheumatol Int* 2017;37:1527-39.

For peer review only - http://bmjopen.bmj.com/site/about/guidelines.xhtml

2. Säll Hansson K, Fridlund B, Brunt D, Hansson B. The meaning of the experiences of persons with chronic pain in their encounter with the health service. *Scand. J. Caring Sci.* 2011;25: 444-50.

3. Kosec, E. (2019). *Physiology of pain and regulation*. In Kosec E, Lampa L, Nisell R. (Red.), Pain and inflammation. Lund: Studentlitteratur.

4. Kosek E, Cohen M, Baron R, Gebhart GF, *et al.* (2016). Do we need a third mechanistic descriptor for chronic pain states? *Pain,*157:1382–6.

5. Jensen KB, Kosek E, Petzke F, Carville S, *et al.* (2009). "Evidence of dysfunctional pain inhibition in Fibromyalgia reflected in rACC during provoked pain." *Pain*, 144(1-2): 95-100.

6. Sluka KA, Clauw DJ. (2016). "Neurobiology of fibromyalgia and chronic widespread pain." *Neuroscience,* 338: 114-129.

7. Gerdle B, Larsson B. (2018). Muscle. Fibromyalgia syndrome and widespread pain – from construction to relevant recognition. In: Häuser W and Perrot S. Philadelphia, Wolters Kluwer: 215- 231.

8. Üçeyler N, Sommer C. (2018). Small nerve fiber pathology. Fibromylagia syndrome and widespread pain - from construction to relevant recognition. In: Häuser W and Perrot S. Philadelphia, Wolters Kluwer: 204-214.

9. Albrecht DS, Forsberg A, Sandstrom A, Bergan C *et al.* (2019). "Brain glial activation in fibromyalgia - A multi-site positron emission tomography investigation." *Brain Behav Immun* 75: 72-83.

10. Werner M. (2019) *Pharmacology.* In Werner M, Bäckryd E. (Red.), Acute and cancer related pain. Pain medicine vol 1. Stockholm Liber.

11. Moore RA, Fisher E, Häuser W, Bell RF, *et al.* (2021). Pharmacological therapies for fibromyalgia (fibromyalgia syndrome) in adults - an overview

For peer review only - http://bmjopen.bmj.com/site/about/guidelines.xhtml

of Cochrane Reviews. *Cochrane Database of Systematic Reviews*, 2021; Issue 8. Art. No.: CD013151.

12. Ming Z, Wenjuan H, Sanjue H, Hui X. Methylcobolamin: A potential Vitamin of Pain Killer. *Neural Plasticity* 2013; Article ID 424651, 6p.

13. Chiu, CK., Low, TH., Tey YS., Singh, VA. & Shong, HK. (2011). The efficacy and safety of intramuscular injections of methylcobalamin in patients with chronic nonspecific low back pain: a randomized controlled trial. *SMJ*, Dec; 52 (12): 868-73.

14. Jurna I. (1998). Analgesic and analgesia-potentiating action of B vitamins. *Schmertz,* Apr, 20;12 (2):136-41.

15. Yu CZ, Liu YP, Lui S, Yan M, *et al.* (2014). Systematic administration of B vitamins attenuates neuropathic hyperalgesia and reduces spinal neuron injury following temporary spinal cord ischemia in rats. *Eur J Pain,*Jan; 18 (1): 76-85.

16. Julian T, Syeed R, Glascow N, Angelopoulou E *et al.* (2020) B12 as a treatment for Peripheral Neurophatic Pain; A Systematic Review. *Nutrients 12*(8), 2221.

17. MC van Campen L, Riepma K, Visser FC. (2019). Open Trial of Vitamin B12 Nasal Drops in Adults With Myalgic Encephalomyelitis/Chronic Fatigue Syndrome: Comparison of Responders and Non-Responders. *Front Pharmacol.* Sep; 20;10:1102

18. Liu HL, Chiu SC. (2015). The effectiveness of vitamin B12 for relieving pain in aphthous ulcers: a randomized, double-blind, placebo controlled trial. *Pain Manag Nurs*. 16 (3), 182-187.

For peer review only - http://bmjopen.bmj.com/site/about/guidelines.xhtml

19. Mibielli MA, Geller M, Cohen JC, Goldberg SG, *et al.* (2019). Diclofenac plus B vitamins versus diclofenac monotherapy in lumbago: the DOLOR study. *Curr Med Res Opin.* Nov; 25(11):2589-99.

20. Yaqub BA, Saddique A, Sulimani R. (1992). Effects of methylcobalamin on diabetic neuropathy. *Clin Neurol Neurosurg*. 94(2), 105-111.

21. Fonseca VA, Lavery L, Thethi T, Daoud Y, *et al.* (2013). Metanx in type 2 diabetes with peripheral neuropathy: a randomized trial. *Am J Med*. 126(2), 141-9.

22. Mauro GL, Martorana U, Cataldo P, Brancato G, *et al.* (2000). Vitamin B12 in low back pain: a randomised, double blind, placebo-controlled study. *Eur Rev Pharmacol Sci*. 4 (3), 53-8.

23. Dahlberg K, Dahlberg H, Nyström M. (2008) *Reflective Lifeworld Research.* Lund: Studentlitteratur.

24. APL´s Summary of Product Characteristics, Product Monograph Mekobalamin https://www.apl.se/samhallsuppdraget/aida.html?sv.target=12.1f169aa2173042d75c421c68&sv.12.1f169aa2173042d75c421c68.route=%2Fproduct%2F176%2Fdocument&returnTo=%7B%22route%22%3A%22%2Ftherapyarea%2F5%2Fproducts%22%7D. (Retrieved 2102125)

25. FASS Natriumklorid https://www.fass.se/LIF/product?userType=0&nplId=19900316000177 (Retrieved 20210225)

26. Buckhardt CS, Bjelle A. A Swedish Version of the Short-Form McGill Pain Questionnaire. *Scand. J. Rheumatol.* 1994;Vol 23, Issue 2

For peer review only - http://bmjopen.bmj.com/site/about/guidelines.xhtml

27. Orwelius L, Nilsson M, Nilsson E, Wenemark M, *et al.* The Swedish RAND-36 health Survey-reliability and responsiveness assessed in patient populations using Svensson´s method for paired ordinal data. *J Patient Rep Outcomes*, 2017;2(1):4.

28. Stening K, Eriksson O, Wahren LK, Berg G, *et al.* Pain sensations to the cold pressor test in normally menstruating women: comparison with men and relation to menstrual phase and serum sex steroid levels. *Am. J. Physiol. Cell Physiol.* 2007;Vol. 293 no. 4.

29. Stening KD, Eriksson O, Henriksson KG, Brynhildsen J, *et al.* Hormonal replacement therapy does not affect self-estimated pain or experimental pain responses in post-menopausal women suffering fibromyalgia a double-blind, randomized, placebo-controlled trial. *Rheumatology* 2011;50;544-55.

30. Walsh NE, Schoenfeld L, Ramamurthy S, Hoffman J. Normative model for cold pressor test. *Am J Phys Med Rehabil* 1989;68:6-11.

31. Ficher AA. Pressure algometry over normal muscles. Standard values, validity and reproducibility of pressure threshold. *Pain* 1987;30:115-126.

32. Rolke R, Andrews Campell K, Magerl W, Treede RD. Deep pain threshold in the distal limb of healthy human subjects. *Eur J Pain* 2005;9: 39-48.

33. Numeric Rating Scale (NRS). https://www.vardhandboken.se/vard-och-behandling/akut-bedomning-och-skattning/smartskattning-av-akut-och-postoperativ-smarta/smartskattningsinstrument/ (Retrieved 20220103).

34. Rampakakis E, Ste-Marie P, Sampalis J, Karellis A, et al. Real-life assesmement of the validity of patient global impression of change in fibromylagia. *RMD Open* 2015;1(1).

For peer review only - http://bmjopen.bmj.com/site/about/guidelines.xhtml

35. Vase EL, Robinson GM, Verne DN, Price DD. (2005). Increased placebo analgesia over time in irritable bowel syndrome (IBS) patients is associated with desire and expectation but not endogenous opioid mechanisms. *Pain*,115:338-47.

36. Vase EL, Robinson GM, Verne DN, Price DD. (2003). The contributions of suggestion, desire, and expectation to placebo effects in irritable bowel syndrome patients: An empirical investigation. *Pain*,105:17-25.

37. Price DD, Barrell JJ. (1984). Some General Laws of Human Emotion - Interrelationships between Intensities of Desire, Expectation, and Emotional Feeling. *J Pers*, 52:389-409.

38. Price DD, Milling LS, Kirsch I, Duff A *et al.* (1999). An analysis of factors that contribute to the magnitude of placebo analgesia in an experimental paradigm. *Pain*, 83:147-56.

39. Kosek E, Rosen A, Carville S, *et al.* (2017). Lower Placebo Responses After Long- Term Exposure to Fibromyalgia Pain. *J Pain*, 18:835-43.

40. The Declaration of Helsinki 2013 https://www.wma.net/policies-post/wma-declaration-of-helsinki-ethical-principles-for-medical-research-involving-human-subjects/ (Retrieved 20220103)

41. Choy E. (2015). *Fibromyalgia Syndrome*, 2nd ed.. Oxford Rheumatology Library. Oxford: University press.

42. Magnusson S, Mannheimer C. (2015) *Long lasting pain. Treatment and rehabilitation*. Lund, Studentlitteratur

43. Cooper S, Gilbert L. An exploratory study of the experience of fibromyalgia diagnosis in *South Africa. Health,* 2017;21(3), 337-353.

44. Fitzcharles MA, Yunus MB. The clinical concept of fibromyalgia as a changing paradigm in the past 20 years. *Pain Res Treat,* 2012: 184835.

For peer review only

For peer review only

For peer review only - http://bmjopen.bmj.com/site/about/guidelines.xhtml

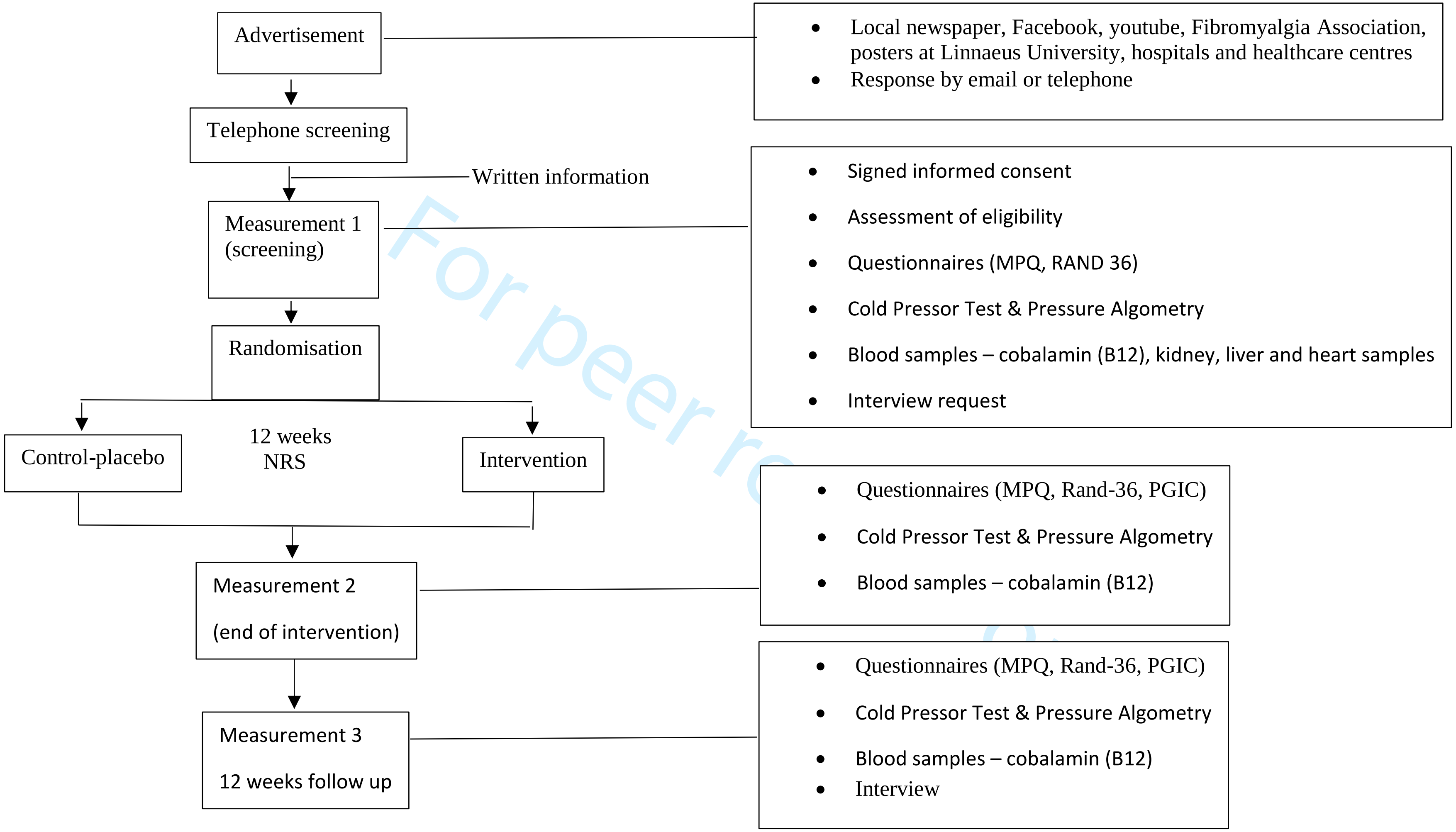

Fig 1. Flowchart of participants, outcome measures and follow-up points

SPIRIT
STANDARD PROTOCOL ITEMS: RECOMMENDATIONS FOR INTERVENTIONAL TRIALS

SPIRIT 2013 Checklist: Recommended items to address in a clinical trial protocol and related documents*

Section/item	Item No	Description
**Administrative information**		
Title	1	Descriptive title identifying the study design, population, interventions, and, if applicable, trial acronym
Trial registration	2a	Trial identifier and registry name. If not yet registered, name of intended registry
	2b	All items from the World Health Organization Trial Registration Data Set
Protocol version	3	Date and version identifier
Funding	4	Sources and types of financial, material, and other support
Roles and responsibilities	5a	Names, affiliations, and roles of protocol contributors
	5b	Name and contact information for the trial sponsor
	5c	Role of study sponsor and funders, if any, in study design; collection, management, analysis, and interpretation of data; writing of the report; and the decision to submit the report for publication, including whether they will have ultimate authority over any of these activities
	5d	Composition, roles, and responsibilities of the coordinating centre, steering committee, endpoint adjudication committee, data management team, and other individuals or groups overseeing the trial, if applicable (see Item 21a for data monitoring committee)
**Introduction**		
Background and rationale	6a	Description of research question and justification for undertaking the trial, including summary of relevant studies (published and unpublished) examining benefits and harms for each intervention
	6b	Explanation for choice of comparators
Objectives	7	Specific objectives or hypotheses
Trial design	8	Description of trial design including type of trial (eg, parallel group, crossover, factorial, single group), allocation ratio, and framework (eg, superiority, equivalence, noninferiority, exploratory)

For peer review only

Methods: Participants, interventions, and outcomes

Study setting	9	Description of study settings (eg, community clinic, academic hospital) and list of countries where data will be collected. Reference to where list of study sites can be obtained
Eligibility criteria	10	Inclusion and exclusion criteria for participants. If applicable, eligibility criteria for study centres and individuals who will perform the interventions (eg, surgeons, psychotherapists)
Interventions	11a	Interventions for each group with sufficient detail to allow replication, including how and when they will be administered
	11b	Criteria for discontinuing or modifying allocated interventions for a given trial participant (eg, drug dose change in response to harms, participant request, or improving/worsening disease)
	11c	Strategies to improve adherence to intervention protocols, and any procedures for monitoring adherence (eg, drug tablet return, laboratory tests)
	11d	Relevant concomitant care and interventions that are permitted or prohibited during the trial
Outcomes	12	Primary, secondary, and other outcomes, including the specific measurement variable (eg, systolic blood pressure), analysis metric (eg, change from baseline, final value, time to event), method of aggregation (eg, median, proportion), and time point for each outcome. Explanation of the clinical relevance of chosen efficacy and harm outcomes is strongly recommended
Participant timeline	13	Time schedule of enrolment, interventions (including any run-ins and washouts), assessments, and visits for participants. A schematic diagram is highly recommended (see Figure)
Sample size	14	Estimated number of participants needed to achieve study objectives and how it was determined, including clinical and statistical assumptions supporting any sample size calculations
Recruitment	15	Strategies for achieving adequate participant enrolment to reach target sample size

Methods: Assignment of interventions (for controlled trials)

Allocation:

Sequence generation	16a	Method of generating the allocation sequence (eg, computer-generated random numbers), and list of any factors for stratification. To reduce predictability of a random sequence, details of any planned restriction (eg, blocking) should be provided in a separate document that is unavailable to those who enrol participants or assign interventions

For peer review only - http://bmjopen.bmj.com/site/about/guidelines.xhtml

Allocation concealment mechanism	16b	Mechanism of implementing the allocation sequence (eg, central telephone; sequentially numbered, opaque, sealed envelopes), describing any steps to conceal the sequence until interventions are assigned
Implementation	16c	Who will generate the allocation sequence, who will enrol participants, and who will assign participants to interventions
Blinding (masking)	17a	Who will be blinded after assignment to interventions (eg, trial participants, care providers, outcome assessors, data analysts), and how
	17b	If blinded, circumstances under which unblinding is permissible, and procedure for revealing a participant's allocated intervention during the trial
**Methods: Data collection, management, and analysis**		
Data collection methods	18a	Plans for assessment and collection of outcome, baseline, and other trial data, including any related processes to promote data quality (eg, duplicate measurements, training of assessors) and a description of study instruments (eg, questionnaires, laboratory tests) along with their reliability and validity, if known. Reference to where data collection forms can be found, if not in the protocol
	18b	Plans to promote participant retention and complete follow-up, including list of any outcome data to be collected for participants who discontinue or deviate from intervention protocols
Data management	19	Plans for data entry, coding, security, and storage, including any related processes to promote data quality (eg, double data entry; range checks for data values). Reference to where details of data management procedures can be found, if not in the protocol
Statistical methods	20a	Statistical methods for analysing primary and secondary outcomes. Reference to where other details of the statistical analysis plan can be found, if not in the protocol
	20b	Methods for any additional analyses (eg, subgroup and adjusted analyses)
	20c	Definition of analysis population relating to protocol non-adherence (eg, as randomised analysis), and any statistical methods to handle missing data (eg, multiple imputation)
**Methods: Monitoring**		
Data monitoring	21a	Composition of data monitoring committee (DMC); summary of its role and reporting structure; statement of whether it is independent from the sponsor and competing interests; and reference to where further details about its charter can be found, if not in the protocol. Alternatively, an explanation of why a DMC is not needed

	21b	Description of any interim analyses and stopping guidelines, including who will have access to these interim results and make the final decision to terminate the trial
Harms	22	Plans for collecting, assessing, reporting, and managing solicited and spontaneously reported adverse events and other unintended effects of trial interventions or trial conduct
Auditing	23	Frequency and procedures for auditing trial conduct, if any, and whether the process will be independent from investigators and the sponsor
**Ethics and dissemination**		
Research ethics approval	24	Plans for seeking research ethics committee/institutional review board (REC/IRB) approval
Protocol amendments	25	Plans for communicating important protocol modifications (eg, changes to eligibility criteria, outcomes, analyses) to relevant parties (eg, investigators, REC/IRBs, trial participants, trial registries, journals, regulators)
Consent or assent	26a	Who will obtain informed consent or assent from potential trial participants or authorised surrogates, and how (see Item 32)
	26b	Additional consent provisions for collection and use of participant data and biological specimens in ancillary studies, if applicable
Confidentiality	27	How personal information about potential and enrolled participants will be collected, shared, and maintained in order to protect confidentiality before, during, and after the trial
Declaration of interests	28	Financial and other competing interests for principal investigators for the overall trial and each study site
Access to data	29	Statement of who will have access to the final trial dataset, and disclosure of contractual agreements that limit such access for investigators
Ancillary and post-trial care	30	Provisions, if any, for ancillary and post-trial care, and for compensation to those who suffer harm from trial participation
Dissemination policy	31a	Plans for investigators and sponsor to communicate trial results to participants, healthcare professionals, the public, and other relevant groups (eg, via publication, reporting in results databases, or other data sharing arrangements), including any publication restrictions
	31b	Authorship eligibility guidelines and any intended use of professional writers
	31c	Plans, if any, for granting public access to the full protocol, participant-level dataset, and statistical code

For peer review only

Appendices

Informed consent materials	32	Model consent form and other related documentation given to participants and authorised surrogates
Biological specimens	33	Plans for collection, laboratory evaluation, and storage of biological specimens for genetic or molecular analysis in the current trial and for future use in ancillary studies, if applicable

*It is strongly recommended that this checklist be read in conjunction with the SPIRIT 2013 Explanation & Elaboration for important clarification on the items. Amendments to the protocol should be tracked and dated. The SPIRIT checklist is copyrighted by the SPIRIT Group under the Creative Commons "Attribution-NonCommercial-NoDerivs 3.0 Unported" license.

For peer review only

BMJ Open

Efficacy of mecobalamin (vitamin B12) in the treatment of long-term pain in women diagnosed with fibromyalgia: protocol for a randomized, placebo-controlled trial

Journal:	*BMJ Open*
Manuscript ID	bmjopen-2022-066987.R1
Article Type:	Protocol
Date Submitted by the Author:	22-Jan-2023
Complete List of Authors:	Sall Hansson, Karin; Linnaeus University Faculty of Health and Life Sciences, Dept. of Health and Caring Sciences, Linnaeus University<br>Lindqvist, Gunilla; Linnaeus University Faculty of Health and Life Sciences, Dept. of Health and Caring Sciences, Linnaeus University<br>Stening, Kent; Linnaeus University Faculty of Health and Life Sciences, Dept. of Health and Caring Sciences, Linnaeus University<br>Fohlman, Jan; Region Kronoberg<br>Wojanowski, Anna; Region Kronoberg<br>Ponten, Moa; Karolinska Institute<br>Jensen, Karin; Karolinska Institute<br>Gerdle, Björn; Linkopings universitet, Pain and Rehabilitation Centre, and Department of Medical and Health Sciences<br>Elmqvist, Carina; Linnaeus University Faculty of Health and Life Sciences, Dept. of Health and Caring Sciences, Linnaeus University; Linnaeus University Faculty of Health and Life Sciences, Centre of Interprofessional Cooperation within Emergency care (CICE)
**Primary Subject Heading**:	Complementary medicine
Secondary Subject Heading:	Health services research, Pharmacology and therapeutics
Keywords:	COMPLEMENTARY MEDICINE, PAIN MANAGEMENT, QUALITATIVE RESEARCH, STATISTICS & RESEARCH METHODS, Clinical trials < THERAPEUTICS

SCHOLARONE™
Manuscripts

Efficacy of mecobalamin (vitamin B12) in the treatment of long-term pain in women diagnosed with fibromyalgia: protocol for a randomized, placebo-controlled trial

Karin Säll Hansson [1 2]
RN, MScN, lecturer, Department of Health and Caring Sciences, Linnaeus University Växjö, Sweden

Gunilla Lindqvist [1 2]
RN, PhD, Senior lecturer, Department of Health and Caring Sciences, Linnaeus University, Växjö, Sweden.

Kent Stening [1 2]
RN, PhD, Senior lecturer, Department of Health and Caring Sciences, Linnaeus University, Kalmar, Sweden

Jan Fohlman [1 2]
MD, PhD, Associate Professor, Region Kronoberg, Sweden

Anna Wojanowski [1 2]
MD, Region Kronoberg, Sweden

Moa Pontén, [3]
MSc, Doctoral student, Department of Clinical Neuroscience, Karolinska Institutet, Sweden

Karin Jensen, [3]
Associate Professor, Department of Clinical Neuroscience, Karolinska Institutet, Sweden

Björn Gerdle, [4]
MD, PhD, Professor emeritus, Department of Health, Medicine and Caring Sciences, Linköping University, Linköping, Sweden

Carina Elmqvist, [1 2]
RN, PhD, Associate Professor, Department of Health and Caring Sciences, Linnaeus University, Växjö, Head of Research, Region Kronoberg, Sweden

[1]Pain Research group, Linnaeus University
[2]Centre of Interprofessional Collaboration within Emergency care (CICE), Linnaeus university
[3]Neuroimagine lab, Karolinska Institute
[4]Pain and Rehabilitation Center, Linkoping, Sweden

Correspondence to:
Carina Elmqvist
Department of Health and Caring Sciences, Linnaeus University, Växjö,

For peer review only

For peer review only - http://bmjopen.bmj.com/site/about/guidelines.xhtml

Head of Research, Region Kronoberg, Sweden
carina.elmqvist@lnu.se

ABSTRACT

Introduction

Fibromyalgia causes long-term pain. It affects at least 2% of the population, the majority being women. In addition, extended symptoms corresponding to vitamin B12 deficiency occur. Findings from several studies have indicated that vitamin B12 may be a possible treatment for pain in fibromyalgia. The aim of the proposed study is to evaluate whether vitamin B12 decreases pain sensitivity and the experience of pain (i.e. hyperalgesia and allodynia) in women with fibromyalgia.

Methods and analysis

The study is a randomized, placebo-controlled, single-blind, clinical trial with two parallel groups who are administered mecobalamin (vitamin B12) or placebo over 12 weeks. 40 Swedish women aged 20-70 years with an earlier recorded diagnosis of fibromyalgia are randomized into the placebo group or the treatment group, each consisting of 20 participants. Outcomes consist of questionnaires measured at baseline and after 12 weeks of treatment. A final re-evaluation will then follow 12 weeks after treatment ends. The primary outcome is tolerance time, maximised to three minutes, which is assessed using the Cold Pressor Test. In order to broaden the understanding of the lived experience of participants, qualitative interviews will be conducted using a phenomenological approach on a life-world theoretical basis (Reflective Lifeworld Research approach).

Ethics and dissemination

The protocol for the study is approved by the local ethical committee at Linkoping (EPM); 2018/294-31 (Appendix 2019-00347; 2020-04482). The principles of the Helsinki Declaration are followed regarding oral and written consent to participate, confidentiality and the possibility to withdraw participation in the study at any time. The results will primarily be communicated through peer-reviewed journals and conferences.

For peer review only - http://bmjopen.bmj.com/site/about/guidelines.xhtml

Trial registration numbers: EudraCT, 2015-005086-23 (Appendix 5.1-2020-71076), protocol no 2020-08-17 version 5.0; ClinicalTrials.gov, NCT05008042.

Keywords: Fibromyalgia, Cobalamin, Chronic pain, Mekobalamin, Randomized controlled trial, Vitamin B12

STRENGTHS AND LIMITATIONS OF THIS STUDY

- The study design is a randomized, placebo-controlled, single-blind clinical trial using parallel groups.
- The trial is a collaboration between different universities and regions.
- A possible limitation is the low number of participants; however, the study continues until 20 participants per group have been included and completed the study, which exceeds the minimum number of 16 determined in our power calculation.
- Another possible limitation is heterogeneity of fibromyalgia spectrum.
- Another possible limitation is the placebo effects of parenteral injection; however, the participants are individually randomized to intervention or control group (placebo) in 1:1 allocation ratio.

For peer review only

INTRODUCTION

Fibromyalgia causes long-term pain and affects at least 2% of the population, 80% of the sufferers being women (1). Treatment options are scarce and patients with fibromyalgia have experienced being discredited and invalidated by the health-care system (2). Fibromyalgia is characterized by long-term widespread musculoskeletal pain and generalized hyperalgesia. This is often accompanied by fatigue, concentration problems and sleep problems (3).

Fibromyalgia pain is currently classified as nociplastic, which means that pain arises from altered regulation of pain signals, in absence of tissue damage (4). No definite pathophysiology has been established for fibromyalgia. Imaging techniques have challenged previous ideas about the

For peer review only - http://bmjopen.bmj.com/site/about/guidelines.xhtml

peripheral origin of FM and have provided evidence for altered central nervous system (CNS) nociceptive/pain processing and morphology in fibromyalgia. Furthermore, recent studies report both central alterations and peripheral alterations (e.g., systemic low-grade inflammation and nociceptor/muscle alterations) (5-9)

Current treatment for fibromyalgia includes both non-pharmacological and pharmacological interventions, depending on the key symptoms and the extent of disability. The recommended pharmacological treatments for fibromyalgia are antidepressants (e.g. SNRI) and antiepileptics (e.g. gabapentinoids), which often cause side effects (4). In addition, opioid analgetics and NSAID/COX are often prescribed (10). Only a minority of individuals report a clinically relevant improvement from any of the treatments (11).

Vitamin B12 is sometimes used for symptoms other than those of vitamin B12 deficiency e.g., different pain conditions such as backpain and neuropathic pain (12-16). Vitamin B12 nasal drops have been tested with positive results on patients with ME/CF/fibromyalgia (myalgic encephalomyelitis/chronic fatigue syndrome) (17). A recent study showed that sublingual vitamin B12 had positive effect on patients with fibromyalgia (18).

In addition, there are vitamin B12 studies that have shown good results in the therapy of aphthous ulcers (19) and acute lumbago (20) as well as in studies concerning diabetic polyneuropathy (21, 22). Moreover, studies examining methylcobalamin treatment in patients with lower back pain showed pain reduction and functionality gain (13, 23).

To summarize, it is not entirely clear how vitamin B12 affects the human pain system. However, several studies indicate that it may be a possible treatment in fibromyalgia.

Aim and objectives

The aim of this study is to evaluate the effect of mecobalamin (vitamin B12), and describe lived experiences of pain, health, suffering and well-being in women with diagnosed fibromyalgia.

For peer review only - http://bmjopen.bmj.com/site/about/guidelines.xhtml

The primary objective is to evaluate whether mecobalamin (vitamin B12) given as an intramuscular injection once/week for 12 weeks compared to placebo reduces pain sensitivity i.e. tolerance time (Cold Pressor Test).

The secondary objective is to evaluate whether intramuscular mecobalamin (vitamin B12) compared to placebo reduces pain intensity and pressure pain threshold (Numeric rating scale NRS, Pressure Algometry), increases activity level (questionnaire), quality of life (questionnaire) and perceived effect of given drug (questionnaire). Furthermore, the ratings of expected effects of a given drug (NRS), the desire for pain relief (NRS) and estimated pain variability (NRS) are evaluated. Qualitative interviews that describe how women with fibromyalgia experience pain, health, suffering and well-being (interviews) will be conducted.

METHODS AND ANALYSIS

Study design

The study is an academic randomized, placebo-controlled, single-blind clinical trial using parallel groups. Participants are individually randomized to intervention or control group (placebo) in 1:1 allocation. In order to broaden and deepen the understanding of the lived experience, an interview will be conducted applying a phenomenological approach on a life-world theoretical basis, Reflective Lifeworld Research approach (RLR) (24).

Study setting and sample

Swedish women aged 20-70 years with an earlier recorded diagnosed fibromyalgia. Detailed eligibility criteria are presented in Table 1.

Table 1. Inclusion and exclusion criteria

**Inclusion criteria:**	**Exclusion criteria:**
- Diagnosis of fibromyalgia<br>- Women aged 20-70 years<br>- Swedish-speaking<br>- Safe method of contraception<br>- Cobalamin value >250 pmol/L < 800 pmol/L	- Previous treatment with B12<br>- Known hypersensitivity to the active substance mecobalamin or an additive<br>- Neuroleptic treatment<br>- Reynaud's phenomenon<br>- Known neuropathy

For peer review only - http://bmjopen.bmj.com/site/about/guidelines.xhtml

- Kidney function value (relatively) GFR >60 mL/min/1.73 m^2<br>- Liver function value P-ALP 0.6-2.85 µkat/L<br>P-ALAT 0.15-1.13 µkat/L<br>- Heart function value Troponin-T < 15 ng/L<br>NT-pro-BNP < 60 years <125 ng/L<br>NT-pro-BNP > 60 years < 300 ng/L<br>- Given consent to participate	- Vegan as veganism can lead to B12 deficiency<br>- Known heart, kidney or liver disease<br>- Breastfeeding<br>- Planned or ongoing pregnancy

The participants are recruited via advertising in the local newspaper Facebook, YouTube, Fibromyalgia Association, posters at Linnaeus University, hospitals and healthcare centres. The prospective participant contacts the Trial Manager via telephone or email and receives oral information. Written information and consent form (Supplemental File) are sent to the participant who contacts the Trial Manager for an appointment.

Intervention and placebo

The active substance of vitamin B12 given in the study is 2ml mecobalamin (5mg/ml) intramuscularly (25). Placebo substance is 2 ml Sodium Chloride (9 mg/ml), isotonic solution for parenteral use (Baxter) intramuscularly (26). All participants are informed that their urine may turn red, which may occur during mecobalamin injections.

Measurements

At the first measurement opportunity, the trial manager and co-examiner 1 carefully follow inclusion and exclusion criteria. Oral information is given again, and consent is signed by the participant and by the co-examiner 1. First, the participant answers two questionnaires Short-form McGill Pain Questionnaire (MPQ) (27) and RAND 36 (28), then stimulation tests (Cold Pressor Test, pressure algometry) (29 - 33) are performed followed by pain rating (NRS) (34) immediately, after one minute and after three minutes.

For peer review only - http://bmjopen.bmj.com/site/about/guidelines.xhtml

Finally, blood samples for cobalamin (vitamin B12), kidney, liver and heart markers are taken. The participant provides an information card about the study's design, purpose, treatment options and contacts with telephone numbers. The participant is asked about interest in an interview in connection with the final measurement opportunity. Co-examiner 2 assesses the blood samples and approves final inclusion. If tests deviate from accepted reference values, the participant is excluded and encouraged to seek medical attention. After approved inclusion, an independent chief examiner performs the randomisation. The participant then receives intramuscular injections of the active substance or placebo, once a week for 12 weeks, given by a registered nurse. Before each injection the participant answers questions in which they estimate their expected effects of a given drug, desire for pain relief and average pain intensity during the past week (NRS). The participant has the opportunity to postpone the visit ± 3 days, for example in case of illness or travelling and may miss a maximum of two injections in order to follow per-protocol analysis. Even if the participant is not compliant with the study protocol, the participant may retain the treatment regime for the duration of the study and be analysed in the main intention-to-treat analysis.

The second measurement takes place after 12 weeks of intervention and three questionnaires (MPQ, RAND 36) and (Patient Global Impression of Change PGIC) (35) are filled out. The stimulation tests Cold Pressor Test and pressure algometry are performed as well as blood sampling of cobalamin/p (vitamin B12). In cases of low cobalamin/p, the participant is encouraged to seek medical attention but is still included in the study.

At the third measurement, which takes place 12 weeks after the end of the intervention, the participant fills in questionnaires (MPQ, RAND-36 and PGIC), undergoes stimulation tests (Cold Pressor Test and pressure algometry), blood sampling of cobalamin/p (vitamin B12) together with an interview. If cobalamin/p on this occasion shows that the participant has a low cobalamin value, the participant is encouraged to seek medical attention. For the individual participant, the study ends after six months. An overview of the study flow is presented in Figure 1.

Outcomes

For peer review only - http://bmjopen.bmj.com/site/about/guidelines.xhtml

The primary outcome is:

- Tolerance time, maximised to three minutes, which is tested using the Cold Pressor Test.

The secondary outcome are:

- Pain experience measured by a pressure algometry test performed on the shoulder, hip, knee and elbow.
- Possible pain change measured by a pressure algometry test performed on the shoulder, hip, knee and elbow.
- Subjective experience of pain measured using Numeric Rating Scale (NRS) 0-10 where 0 is the best outcome.
- Possible pain change measured using Numeric Rating Scale (NRS) 0-10 where 0 is the best outcome.
- Ratings of expectation, desire for relief, using Numeric Rating Scale (NRS) 0-10 where 0 is the best outcome.
- Ratings of expectation, pain variability, using Numeric Rating Scale (NRS) 0-10 where 0 is the best outcome.
- Activity level are assessed using questionnaire McGills Pain Questionnaire (MPQ). Short version score 0-45.
- Quality of life are assessed using questionnaires RAND-36 score 0-100.
- Experience of the effect of the drug, assessed using questionnaire Patients´ Global Impression of Change (PGIC) score 1-7.
- Control of vitamin B12 is done by measuring cobalamin in plasma.
- Qualitative in-depth interviews will be conducted to capture women's lived experiences of pain, health, suffering and well-being.

Cold Pressor Test

The Cold Pressor Test (29-31) is measured in number of seconds the participant leaves her hand in the low-temperature water of five degrees. Evaluation variables from Cold Pressor Test become pain threshold (when it starts to hurt) and tolerance time, i.e. how long the participant endures the

For peer review only - http://bmjopen.bmj.com/site/about/guidelines.xhtml

pain. Participants end the test by raising their hands when they can no longer stand it or when the set end time of three minutes has elapsed.

Pressure algometry test

The pressure algometry test (32, 33) generates a mechanical pressure against specific points on the body; trapezius (shoulder), epicondyle (elbow), gluteal (outside the gluteal muscles) and medial knee (inside of the knee) which are all accepted in the diagnosis of fibromyalgia. Pressure algometry tests are measured in kilopascal (kPa). The Trial Manager interrupts the mechanical pressure when the participant expresses pain.

Numeric rating scale (NRS)

Participants rate their pain intensity on NRS (34) in connection with the Cold Pressor Test when the participant takes her hand from the water, after one minute and finally after three minutes. Participants estimate their pain intensity on a scale (NRS) from 0-10, where 0 corresponds to no pain at all and 10 worst possible pain. Pain rating is measured in the same way after the pressure algometry test.

The influence of expectation, desire for pain relief, and pain variability will be measured before each injection using the same numeric rating scale as used for pain ratings. Ratings of expected pain levels will be obtained by asking patients “What level of pain do you expect when this treatment starts to have an effect?” The NRS scale is anchored at the left by the descriptors ´no pain sensation´ and at the right by ´the most pain sensation imaginable´. Ratings of desired pain relief will be obtained by asking patients “How strong is your desire for pain relief? The question will be anchored by the descriptors ‘0=No desire for pain relief’ and ‘10=the most intense desire for pain relief imaginable’. Ratings of pain variability will be obtained by asking participants their average pain intensity that week. These scales have been validated and used in previous studies (36-40).

Short-form McGill Pain Questionnaire (MPQ)

In this study MPQ Short form of 15 questions (27) is used which distinguishes between the sensory-discriminatory and the affective and

For peer review only - http://bmjopen.bmj.com/site/about/guidelines.xhtml

emotional aspects of the pain experience. Participants are asked to estimate their pain intensity and describe their pain in predetermined words. They fill in MPQ on all three measurement occasions.

RAND-36

RAND-36 is a quality-of-life instrument (28) that aims to measure health-related quality of life, i.e. physical, mental and social well-being, not just the absence of illness. The estimation instrument consists of 36 questions of various kinds, e.g. on general health, activity, physical health, emotional problems and pain. RAND-36 is filled in by the participant on all three measurement occasions.

Patient Global Impression of Change (PGIC)

PGIC (35) evaluates participants' experience of the treatment i.e. the substance given. Participants tick the box that most closely describes the change that they are experiencing. The PGIC is completed by the participant after the injection treatment has been concluded at 12 weeks and at the follow-up 12 weeks later. PGIC is directly translated in its entirety from English by the research group.

Cobalamin/p

Cobalamin/p levels are taken on three occasions. The first measurement, baseline rule out a vitamin B12 deficiency (according to the accepted reference values). A second measurement is performed at 12 weeks on completion of treatment in order to monitor the participants' level of cobalamin/p just after the end of the injection treatment. The final measurement is12 weeks after the end of intervention.

Qualitative in-depth interview

In order to obtain a deeper understanding of women's perceived experiences, in-depth interviews with both compliant and non-compliant participants from both groups will be performed in connection with the third measurement session. The aim is to describe the women's lived experiences of pain,

For peer review only - http://bmjopen.bmj.com/site/about/guidelines.xhtml

health, suffering and well-being. The interviews will be conducted by the Trial Manager, recorded, transcribed and later analysed using a reflective life world approach (RLR) (24).

Sample size

In order to obtain an adequate sample size, a power analysis was performed based on results from a previously published study (29) in which women with fibromyalgia underwent the same stimulation procedure with the Cold Pressor Test. Account is taken here of the standard deviation shown and compensation for any placebo effects. Based on these results, the expected effect difference is estimated at 20 seconds with a standard deviation of 20 seconds between the two groups after 12 weeks of treatment with mecobalamin (vitamin B12)/placebo. Using a two-sided t-test for two independent groups, with a power of 80% and 5% significance level, it was calculated that each group would consist of 16 participants. In order to compensate for any loss, 20 participants per group are therefore included. The study continues until 40 participants have been included and completed the study.

Randomization and allocation concealment

Participants are randomized into two groups, the placebo group or the treatment group, each consisting of 20 participants either receiving mecobalamin (vitamin B12) or placebo. An independent statistician generates a randomisation list by random distribution of processing in blocks (1:1) in a computerised statistics program (STATA). Participants are randomized according to the list by the chief examiner opening closed randomisation envelopes in consecutive order. Based on each randomisation envelope, the chief examiner registers in and signs the injection retrieval protocol as to which substance the participant will receive. The participants' randomisation number is recorded in the journal. Only the chief examiner has access to randomisation envelopes and injection retrieval protocols which are kept locked.

When the third measurement session has been completed, the trial manager contacts the chief examiner who has the randomisation list.

For peer review only - http://bmjopen.bmj.com/site/about/guidelines.xhtml

The chief examiner breaks the code and notifies the participant with a letter of given treatment. The code is broken at this time because it cannot be considered ethical that the participant does not find out about the treatment given, especially if the participant believes that the treatment has helped. If the participant has received mecobalamin and wishes to continue, they are recommended to contact their health centre for continued mecobalamin treatment. The letter will contain the contact information of the trial manager and the chief examiner if the health centre doctor wishes to contact them. The trial manager is still unaware of what the individual participant received until the study is completed. If the code needs to be broken for an individual participant before the third measurement session has been completed, there is a code breaking envelope that may be used by the chief examiner in cases of emergency.

Adverse events (AEs) and serious adverse events (SAEs)

AEs are defined as any unfavourable or unintended reaction occurring in a research person during the course of the study and where investigational medicinal products have been administered, regardless of dose. Any AE will be reported from day 1 of the study at each visit to the clinic and until the last visit made 24 weeks after the start of the study. All SAEs that occur during the course of the study will be reported to the sponsor within 24 hours of the research staff becoming aware of the incident. During the study, an annual safety report from the sponsor and responsible investigator is sent in to both the Swedish Medical Products Agency and the Regional Ethical Review Board.

Statistical methods

The primary endpoint is tolerance time at Cold Pressor Test and it will be calculated on the difference between the two groups after 12 weeks and will be analysed by ANOVA. Pain thresholds measured by pressure algometry test (Somedic) will be analysed using: t-test, ANOVA, similar to the primary variable. Questionnaires (MPQ, RAND-36, and PGIC) will be analysed using Mann-Whitney tests. In cases of minor loss, (less than 10%), a mixed model can be used as an alternative to t-test and ANOVA with repeated

For peer review only - http://bmjopen.bmj.com/site/about/guidelines.xhtml

measurement. For variables with more than 10% loss, imputation of data will be used. Drop-out participants will be described in a specific analysis. Rejection analysis will be performed on the participants who interrupt the study prematurely.

Results will be reported for both intention-to-treat and per-protocol analyses. Intention-to-treat is defined as the participant is placed in the treatment group she had been randomized to. Per-protocol is defined as the participant is placed in the treatment group she de facto received and carried out without significant protocol deviations. The per-protocol results will be presented as sensitivity analyses. The participant may miss a maximum of two injections.

Self-reported ratings of desired and expected treatment outcome pre and post treatment will be implemented in linear regression models to predict any of the outcome measures. Repeated ratings of desired treatment outcome, expected treatment outcome and pain variability will be calculated as each participant's standard deviation from the repeated ratings and implemented in a linear regression model to predict possible outcome measures.

Patient and public involvement

None.

ETHICS AND DISSEMINATION

The study is registered at the Swedish Medical Products Agency, EudraCT-no 2015-005086-23 (Appendix 5.1-2020-71076), protocol no 2020-08-17 version 5.0 and ClinicalTrials.gov (NCT05008042). The trial was approved by the local ethical committee at Linkoping (EPM); 2018/294-31 (Appendix 2019-00347; 2020-04482). The principles of the Helsinki Declaration (2013) (41) are followed regarding oral and written consent to participate, confidentiality and the possibility to withdraw participation in the study at any time. Good Clinical Practice (GCP) is followed, with independent regular monitoring by Forum Ostergotland, Linkopings University, including plans for communicating important protocol modifications. The data will be placed in a locked cabinet at the university until the study is completed and

For peer review only - http://bmjopen.bmj.com/site/about/guidelines.xhtml

the data is to be processed and analysed. Only involved researchers have access to data when it is to be analysed; however, anyone requesting metadata can access the data during 2025-2035. The study started on February 6th, 2019, but recruitment was delayed due to the COVID-19 pandemic. Final recruitment is expected to be completed in 2024. The results will primarily be communicated through peer-reviewed journals and conferences.

*** ***

Contributors

KSH (trial manager), GL, KS, JF (chief examiner), MP, KJ, BG and CE (sponsor) planned the study design and all authors participated in the production of the analysis plan. KSH, GL, KS, AWo and CE prepared the first version of the manuscript and all authors participated in reviewing and drafting the manuscript. All authors have read and approved the final version.

Competing interests

There are no conflicts of interest to report.

Funding

This trial was supported by grants from Medical Research Council of Southeast Sweden no: 479961; 656241; 854911 and Capio Research Foundation, Sweden no: 2018-3195; 2019-3277. The funding sources had no involvement in study design, data collection or the writing of this manuscript.

Acknowledgements

We thank Co-examiner and Medical Doctors; Anders Willstedt and Bjarne Sorensen as well as the Registered Nurses Ann-Louise Breider and Lisa Gunnarsson for their ongoing contributions and support.

For peer review only - http://bmjopen.bmj.com/site/about/guidelines.xhtml

REFERENCES

1. Heidari F, Afshari M, Moosazadeh M. Prevalence of fibromyalgia in general population and patients, a systematic review and meta-analysis. *Rheumatol Int* 2017;37:1527-39.

2. Säll Hansson K, Fridlund B, Brunt D, *et al.* The meaning of the experiences of persons with chronic pain in their encounter with the health service. *Scand. J. Caring Sci.* 2011;25:444-50.

3. Kosec, E. (2019). *Physiology of pain and regulation*. In Kosec E, Lampa L, Nisell R. (Red.), Pain and inflammation. Lund: Studentlitteratur.

4. Kosek E, Cohen M, Baron R, *et al.* Do we need a third mechanistic descriptor for chronic pain states? *Pain,* 2016;157:1382–6.

5. Jensen KB, Kosek E, Petzke F, *et al.* "Evidence of dysfunctional pain inhibition in Fibromyalgia reflected in rACC during provoked pain." *Pain*, 2009;144(1-2): 95-100.

6. Sluka KA, Clauw DJ. "Neurobiology of fibromyalgia and chronic widespread pain." *Neuroscience,* 2016;338:114-129.

7. Gerdle B, Larsson B. (2018). Muscle. Fibromyalgia syndrome and widespread pain – from construction to relevant recognition. In: Häuser W and Perrot S. Philadelphia, Wolters Kluwer: 215- 231.

8. Üçeyler N, Sommer C. (2018). Small nerve fiber pathology. Fibromylagia syndrome and widespread pain - from construction to relevant recognition. In: Häuser W and Perrot S. Philadelphia, Wolters Kluwer: 204-214.

9. Albrecht DS, Forsberg A, Sandstrom A, *et al.* "Brain glial activation in fibromyalgia - A multi-site positron emission tomography investigation." *Brain Behav Immun* 2019;75:72-83.

For peer review only - http://bmjopen.bmj.com/site/about/guidelines.xhtml

10. Werner M. (2019) *Pharmacology.* In Werner M, Bäckryd E. (Red.), Acute and cancer related pain. Pain medicine vol 1. Stockholm Liber.

11. Moore RA, Fisher E, Häuser W, *et al.* Pharmacological therapies for fibromyalgia (fibromyalgia syndrome) in adults - an overview of Cochrane Reviews. *Cochrane Database of Systematic Reviews*, 2021; (8).

12. Ming Z, Wenjuan H, Sanjue H, *et al.* Methylcobalamin: A potential Vitamin of Pain Killer. *Neural Plast,* 2013; 2013:424651.

13. Chiu CK, Low TH, Tey YS, *et al.* The efficacy and safety of intramuscular injections of methylcobalamin in patients with chronic nonspecific low back pain: a randomized controlled trial. *SMJ*, 2011; 52 (12): 868-73.

14. Jurna I. Analgesic and analgesia-potentiating action of B vitamins. *Schmertz,* 1998;20;12(2):136-41.

15. Yu CZ, Liu YP, Lui S, *et al.* Systematic administration of B vitamins attenuates neuropathic hyperalgesia and reduces spinal neuron injury following temporary spinal cord ischemia in rats. *Eur J Pain,*Jan; 2014;18(1):76-85.

16. Julian T, Syeed R, Glascow N, *et al.*
B12 as a treatment for Peripheral Neurophatic Pain; A Systematic Review. *Nutrients*, 2020;*12*(8):2221.

17. MC van Campen L, Riepma K, Visser FC. Open Trial of Vitamin B12 Nasal Drops in Adults With Myalgic Encephalomyelitis/Chronic Fatigue Syndrome: Comparison of Responders and Non-Responders. *Front Pharmacol.* 2019;20(10):1102

For peer review only - http://bmjopen.bmj.com/site/about/guidelines.xhtml

18. Gharibpoor F, Gavidel-Parsa B, Sattari N, *et al.* Effect of vitamin B12 on the symptom severity and psychological profile of fibromyalgia patients; a prospective pre-post study.
BMC Rheuma. 2022;51(6).

19. Liu HL, Chiu SC. The effectiveness of vitamin B12 for relieving pain in aphthous ulcers: a randomized, double-blind, placebo controlled trial. *Pain Manag Nurs*. 2015;16(3):182-187.

20. Mibielli MA, Geller M, Cohen JC, *et al.* Diclofenac plus B vitamins versus diclofenac monotherapy in lumbago: the DOLOR study. *Curr Med Res Opin.* 2019;25(11):2589-99.

21. Yaqub BA, Saddique A, Sulimani R. Effects of methylcobalamin on diabetic neuropathy. *Clin Neurol Neurosurg*. 1992;94(2):105-111.

22. Fonseca VA, Lavery L, Thethi T, *et al.* Metanx in type 2 diabetes with peripheral neuropathy: a randomized trial. *Am J Med*. 2013;126(2):141-9.

23. Mauro GL, Martorana U, Cataldo P, *et al.* Vitamin B12 in low back pain: a randomized, double blind, placebo-controlled study. *Eur Rev Pharmacol Sci*. 2000;4(3):53-8.

24. Dahlberg K, Dahlberg H, Nyström M. (2008) *Reflective Lifeworld Research.* Lund: Studentlitteratur.

25. APL´s Summary of Product Characteristics, Product Monograph Mekobalamin
https://www.apl.se/samhallsuppdraget/aida.html?sv.target=12.1f169aa2173042d75c421c68&sv.12.1f169aa2173042d75c421c68.route=%2Fproduct%2F176%2Fdocument&returnTo=%7B%22route%22%3A%22%2Ftherapyarea%2F5%2Fproducts%22%7D. (Retrieved 2102125)

For peer review only - http://bmjopen.bmj.com/site/about/guidelines.xhtml

26. FASS Natriumklorid https://www.fass.se/LIF/product?userType=0&nplId=19900316000177 (Retrieved 20210225)

27. Buckhardt CS, Bjelle A. A Swedish Version of the Short-Form McGill Pain Questionnaire. *Scand. J. Rheumatol.* 1994;23(2).

28. Orwelius L, Nilsson M, Nilsson E, *et al.* The Swedish RAND-36 health Survey-reliability and responsiveness assessed in patient populations using Svensson´s method for paired ordinal data. *J Patient Rep Outcomes*, 2017;2(1):4.

29. Stening K, Eriksson O, Wahren LK, *et al.* Pain sensations to the cold pressor test in normally menstruating women: comparison with men and relation to menstrual phase and serum sex steroid levels. *Am. J. Physiol. Cell Physiol.* 2007;293(4).

30. Stening KD, Eriksson O, Henriksson KG, *et al.* Hormonal replacement therapy does not affect self-estimated pain or experimental pain responses in post-menopausal women suffering fibromyalgia a double-blind, randomized, placebo-controlled trial. *Rheumatology* 2011;50:544-55.

31. Walsh NE, Schoenfeld L, Ramamurthy S, *et al.* Normative model for cold pressor test. *Am J Phys Med Rehabil* 1989;68:6-11.

32. Ficher AA. Pressure algometry over normal muscles. Standard values, validity and reproducibility of pressure threshold. *Pain* 1987;30:115-126.

33. Rolke R, Andrews Campell K, Magerl W, *et al.* Deep pain threshold in the distal limb of healthy human subjects. *Eur J Pain* 2005;9:39-48.

For peer review only - http://bmjopen.bmj.com/site/about/guidelines.xhtml

34. Numeric Rating Scale (NRS). https://www.vardhandboken.se/vard-och-behandling/akut-bedomning-och-skattning/smartskattning-av-akut-och-postoperativ-smarta/smartskattningsinstrument/ (Retrieved 20220103).

35. Rampakakis E, Ste-Marie P, Sampalis J, *et al*. Real-life assesmement of the validity of patient global impression of change in fibromylagia. *RMD Open,* 2015;1(1).

36. Vase EL, Robinson GM, Verne DN, *et al.* Increased placebo analgesia over time in irritable bowel syndrome (IBS) patients is associated with desire and expectation but not endogenous opioid mechanisms. *Pain*, 2005;115:338-47.

37. Vase EL, Robinson GM, Verne DN, *et al.* The contributions of suggestion, desire, and expectation to placebo effects in irritable bowel syndrome patients: An empirical investigation. *Pain*, 2003;105:17-25.

38. Price DD, Barrell JJ. Some General Laws of Human Emotion - Interrelationships between Intensities of Desire, Expectation, and Emotional Feeling. *J Pers*, 1984;52:389-409.

39. Price DD, Milling LS, Kirsch I, *et al.* An analysis of factors that contribute to the magnitude of placebo analgesia in an experimental paradigm. *Pain*, 1999;83:147-56.

40. Kosek E, Rosen A, Carville S, *et al.* Lower Placebo Responses After Long- Term Exposure to Fibromyalgia Pain. *J Pain*, 2017;18:835-43.

41. The Declaration of Helsinki 2013 https://www.wma.net/policies-post/wma-declaration-of-helsinki-ethical-principles-for-medical-research-involving-human-subjects/ (Retrieved 20220103)

Figure title

For peer review only - http://bmjopen.bmj.com/site/about/guidelines.xhtml

Figure 1. Flowchart of participants, outcome measures and follow-up points

For peer review only

For peer review only - http://bmjopen.bmj.com/site/about/guidelines.xhtml

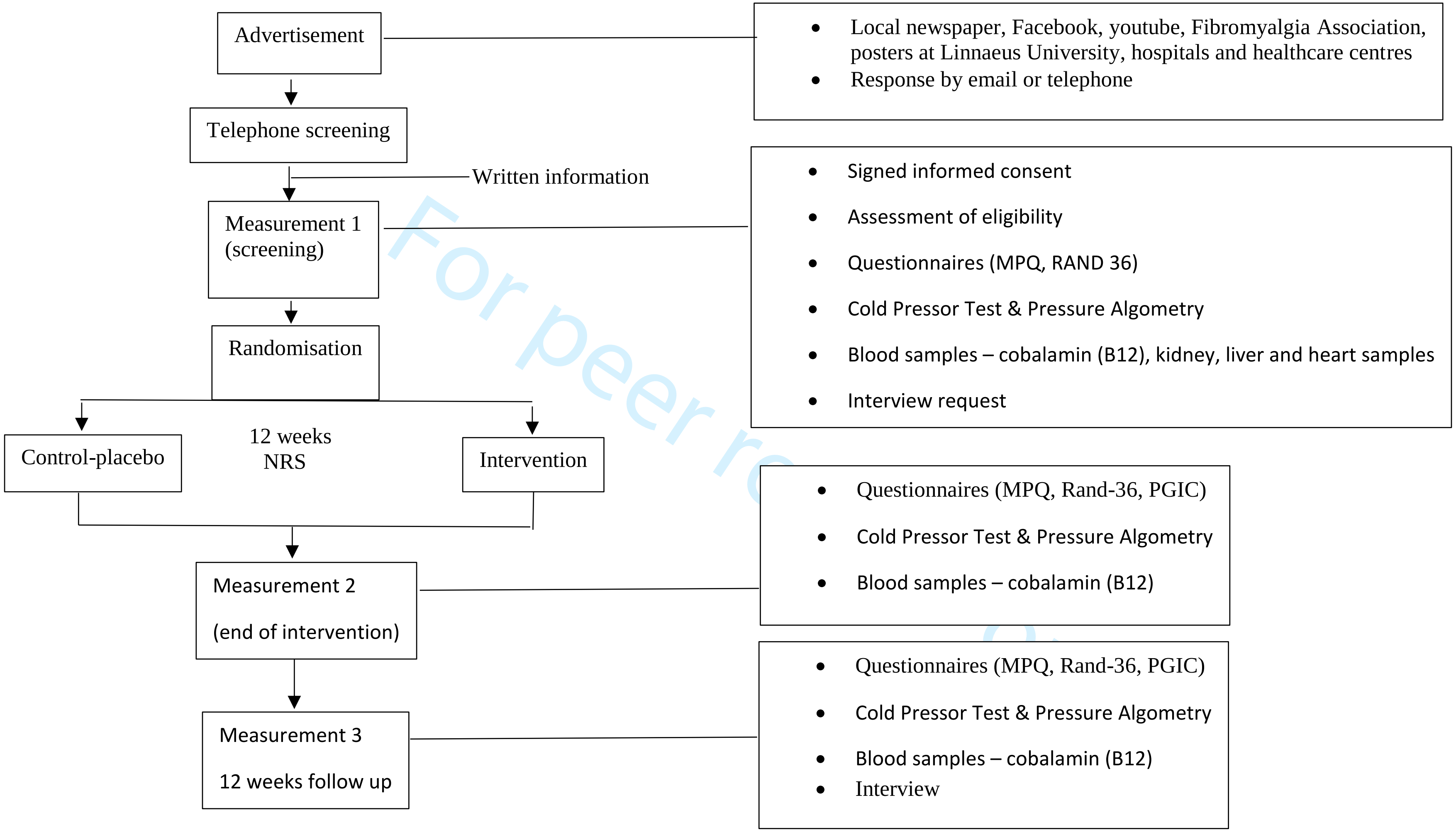

Fig 1. Flowchart of participants, outcome measures and follow-up points

Participant informed consent form

I have been informed orally about the study and I have read the written information. I have received answers to my questions and I agree to participate in the study. My participation is voluntary, and I am aware that I can withdraw without any explanation and without affecting my future care.

I am informed about my rights to get register extract once a year over collected data. I have been informed about my rights to get false information's arranged or removed from the register. I have been informed about samples taken in the study are handled according to the Biobank Act. I am aware that my sample material will be destroyed after analyses.

Consent regarding data management

I have received information that personal data collected in the study will be handled confidentially, which means my identity will not be revealed to unauthorized persons.

I allow an independent person and/or authority person, appointed by the study management, to review study information provided customary confidentiality are maintained.

We ask you to confirm your identity at all occasions with legitimation.

……………………………. ………………………………….

Participants' signature Date (written by participant)

……………………………..

Name clarification

………………………………. …………………………………

Physician signature Date

…………………………………

Name clarification

☐ Signed copy of the information and consent has been distributed to the participant.

For peer review only - http://bmjopen.bmj.com/site/about/guidelines.xhtml

For peer review only

For peer review only - http://bmjopen.bmj.com/site/about/guidelines.xhtml

SPIRIT

STANDARD PROTOCOL ITEMS: RECOMMENDATIONS FOR INTERVENTIONAL TRIALS

SPIRIT 2013 Checklist: Recommended items to address in a clinical trial protocol and related documents*

Section/item	Item No	Description	Addressed on page number
**Administrative information**			
Title	1	Descriptive title identifying the study design, population, interventions, and, if applicable, trial acronym	Page 1
Trial registration	2a	Trial identifier and registry name. If not yet registered, name of intended registry	Page 3 and 14
	2b	All items from the World Health Organization Trial Registration Data Set	______________
Protocol version	3	Date and version identifier	Page 3 and 14
Funding	4	Sources and types of financial, material, and other support	Page 15
Roles and responsibilities	5a	Names, affiliations, and roles of protocol contributors	Page 15
	5b	Name and contact information for the trial sponsor	Page 1
	5c	Role of study sponsor and funders, if any, in study design; collection, management, analysis, and interpretation of data; writing of the report; and the decision to submit the report for publication, including whether they will have ultimate authority over any of these activities	Page 15
	5d	Composition, roles, and responsibilities of the coordinating centre, steering committee, endpoint adjudication committee, data management team, and other individuals or groups overseeing the trial, if applicable (see Item 21a for data monitoring committee)	Page 1 and 15

For peer review only

**Introduction**			
Background and rationale	6a	Description of research question and justification for undertaking the trial, including summary of relevant studies (published and unpublished) examining benefits and harms for each intervention	Page 3-5
	6b	Explanation for choice of comparators	
Objectives	7	Specific objectives or hypotheses	Page 5
Trial design	8	Description of trial design including type of trial (eg, parallel group, crossover, factorial, single group), allocation ratio, and framework (eg, superiority, equivalence, noninferiority, exploratory)	Page 5
**Methods: Participants, interventions, and outcomes**			
Study setting	9	Description of study settings (eg, community clinic, academic hospital) and list of countries where data will be collected. Reference to where list of study sites can be obtained	Page 6
Eligibility criteria	10	Inclusion and exclusion criteria for participants. If applicable, eligibility criteria for study centres and individuals who will perform the interventions (eg, surgeons, psychotherapists)	Page 5
Interventions	11a	Interventions for each group with sufficient detail to allow replication, including how and when they will be administered	Page 6-8
	11b	Criteria for discontinuing or modifying allocated interventions for a given trial participant (eg, drug dose change in response to harms, participant request, or improving/worsening disease)	Page 6-8
	11c	Strategies to improve adherence to intervention protocols, and any procedures for monitoring adherence (eg, drug tablet return, laboratory tests)	Page 6-8
	11d	Relevant concomitant care and interventions that are permitted or prohibited during the trial	
Outcomes	12	Primary, secondary, and other outcomes, including the specific measurement variable (eg, systolic blood pressure), analysis metric (eg, change from baseline, final value, time to event), method of aggregation (eg, median, proportion), and time point for each outcome. Explanation of the clinical relevance of chosen efficacy and harm outcomes is strongly recommended	Page 8 - 11
Participant timeline	13	Time schedule of enrolment, interventions (including any run-ins and washouts), assessments, and visits for participants. A schematic diagram is highly recommended (see Figure)	Page 6-8 - fig 1

For peer review only - http://bmjopen.bmj.com/site/about/guidelines.xhtml

Sample size	14	Estimated number of participants needed to achieve study objectives and how it was determined, including clinical and statistical assumptions supporting any sample size calculations	Page 12
Recruitment	15	Strategies for achieving adequate participant enrolment to reach target sample size	Page 6

Methods: Assignment of interventions (for controlled trials)

Allocation:

Sequence generation	16a	Method of generating the allocation sequence (eg, computer-generated random numbers), and list of any factors for stratification. To reduce predictability of a random sequence, details of any planned restriction (eg, blocking) should be provided in a separate document that is unavailable to those who enrol participants or assign interventions	Page 12
Allocation concealment mechanism	16b	Mechanism of implementing the allocation sequence (eg, central telephone; sequentially numbered, opaque, sealed envelopes), describing any steps to conceal the sequence until interventions are assigned	Page 12
Implementation	16c	Who will generate the allocation sequence, who will enrol participants, and who will assign participants to interventions	Page 12
Blinding (masking)	17a	Who will be blinded after assignment to interventions (eg, trial participants, care providers, outcome assessors, data analysts), and how	Page 12
	17b	If blinded, circumstances under which unblinding is permissible, and procedure for revealing a participant's allocated intervention during the trial	Page 12

Methods: Data collection, management, and analysis

Data collection methods	18a	Plans for assessment and collection of outcome, baseline, and other trial data, including any related processes to promote data quality (eg, duplicate measurements, training of assessors) and a description of study instruments (eg, questionnaires, laboratory tests) along with their reliability and validity, if known. Reference to where data collection forms can be found, if not in the protocol	Page 9-11
	18b	Plans to promote participant retention and complete follow-up, including list of any outcome data to be collected for participants who discontinue or deviate from intervention protocols	Page 13

For peer review only - http://bmjopen.bmj.com/site/about/guidelines.xhtml

Data management	19	Plans for data entry, coding, security, and storage, including any related processes to promote data quality (eg, double data entry; range checks for data values). Reference to where details of data management procedures can be found, if not in the protocol	Page 12 -14
Statistical methods	20a	Statistical methods for analysing primary and secondary outcomes. Reference to where other details of the statistical analysis plan can be found, if not in the protocol	Page 13-14
	20b	Methods for any additional analyses (eg, subgroup and adjusted analyses)	Page 13-14
	20c	Definition of analysis population relating to protocol non-adherence (eg, as randomised analysis), and any statistical methods to handle missing data (eg, multiple imputation)	Page 13-14
**Methods: Monitoring**			
Data monitoring	21a	Composition of data monitoring committee (DMC); summary of its role and reporting structure; statement of whether it is independent from the sponsor and competing interests; and reference to where further details about its charter can be found, if not in the protocol. Alternatively, an explanation of why a DMC is not needed	Page 14
	21b	Description of any interim analyses and stopping guidelines, including who will have access to these interim results and make the final decision to terminate the trial	______________
Harms	22	Plans for collecting, assessing, reporting, and managing solicited and spontaneously reported adverse events and other unintended effects of trial interventions or trial conduct	Page 13
Auditing	23	Frequency and procedures for auditing trial conduct, if any, and whether the process will be independent from investigators and the sponsor	Page 14
**Ethics and dissemination**			
Research ethics approval	24	Plans for seeking research ethics committee/institutional review board (REC/IRB) approval	Page 14
Protocol amendments	25	Plans for communicating important protocol modifications (eg, changes to eligibility criteria, outcomes, analyses) to relevant parties (eg, investigators, REC/IRBs, trial participants, trial registries, journals, regulators)	Page 14

For peer review only - http://bmjopen.bmj.com/site/about/guidelines.xhtml

Consent or assent	26a	Who will obtain informed consent or assent from potential trial participants or authorised surrogates, and how (see Item 32)	Page 7 and 14
	26b	Additional consent provisions for collection and use of participant data and biological specimens in ancillary studies, if applicable	
Confidentiality	27	How personal information about potential and enrolled participants will be collected, shared, and maintained in order to protect confidentiality before, during, and after the trial	Page 12 and 14
Declaration of interests	28	Financial and other competing interests for principal investigators for the overall trial and each study site	Page 15
Access to data	29	Statement of who will have access to the final trial dataset, and disclosure of contractual agreements that limit such access for investigators	Page 14
Ancillary and post-trial care	30	Provisions, if any, for ancillary and post-trial care, and for compensation to those who suffer harm from trial participation	
Dissemination policy	31a	Plans for investigators and sponsor to communicate trial results to participants, healthcare professionals, the public, and other relevant groups (eg, via publication, reporting in results databases, or other data sharing arrangements), including any publication restrictions	Page 15
	31b	Authorship eligibility guidelines and any intended use of professional writers	
	31c	Plans, if any, for granting public access to the full protocol, participant-level dataset, and statistical code	Page 14
**Appendices**			
Informed consent materials	32	Model consent form and other related documentation given to participants and authorised surrogates	Page 7 and14
Biological specimens	33	Plans for collection, laboratory evaluation, and storage of biological specimens for genetic or molecular analysis in the current trial and for future use in ancillary studies, if applicable	

*It is strongly recommended that this checklist be read in conjunction with the SPIRIT 2013 Explanation & Elaboration for important clarification on the items. Amendments to the protocol should be tracked and dated. The SPIRIT checklist is copyrighted by the SPIRIT Group under the Creative Commons "Attribution-NonCommercial-NoDerivs 3.0 Unported" license.

For peer review only - http://bmjopen.bmj.com/site/about/guidelines.xhtml
